# Clinical presentation and symptomatology of Guillain-Barré syndrome: A literature review

**DOI:** 10.1097/MD.0000000000038890

**Published:** 2024-07-26

**Authors:** Chukwuka Elendu, Emmanuella I. Osamuyi, Ikeoluwa A. Afolayan, Nnamdi C. Opara, Nkeiruka A. Chinedu-Anunaso, Chinonso B. Okoro, Augustine U. Nwankwo, Dianne O. Ezidiegwu, Chinweike A. Anunaso, Collins C. Ogbu, Samuel O. Aghahowa, Chibuzor S. Atuchukwu, Everister U. Akpa, Jesse C. Peterson

**Affiliations:** a Federal University Teaching Hospital, Owerri, Nigeria; b Bingham University Teaching Hospital, Jos, Nigeria; c Plateaumed Nigeria Limited, Lagos, Nigeria; d Imo State University, Owerri, Nigeria; e University of Nigeria Teaching Hospital, Ituku-Ozalla, Nigeria; f Nnamdi Azikiwe University Teaching Hospital Nnewi, Nnewi, Nigeria; g Alex Ekwueme Federal University Ndufu-Alike, Abakaliki, Nigeria; h University of Port Harcourt Teaching Hospital, Choba, Nigeria; i Nigerian Navy Reference Hospital, Ojo, Nigeria; j Jos University Teaching Hospital, Jos, Nigeria.

**Keywords:** Guillain-Barré Syndrome (GBS), immunomodulatory therapies, multidisciplinary approach, neurological disorder, rehabilitation

## Abstract

Guillain-Barré Syndrome (GBS) is a rare but potentially life-threatening neurological disorder characterized by acute onset ascending paralysis and sensory abnormalities. This article provides a comprehensive overview of GBS, covering its epidemiology, etiology, clinical presentation, diagnostic evaluation, management and treatment, prognosis, psychosocial impact, recent advances in research, public health implications, and ethical considerations. Epidemiological data reveal variations in GBS prevalence, incidence rates, and geographical distribution influenced by climate, infectious disease prevalence, and genetic susceptibility. Etiological factors include preceding infections, vaccinations, and autoimmune mechanisms, although the precise pathophysiology remains incomplete. Clinical presentation encompasses prodromal symptoms, motor deficits, sensory abnormalities, autonomic dysfunction, and variants such as Miller-Fisher Syndrome and Bickerstaff brainstem encephalitis. Neurological examination findings include weakness, paralysis, sensory deficits, and reflex changes, while autonomic dysfunction manifests as cardiovascular, respiratory, and gastrointestinal symptoms. Diagnostic evaluation relies on clinical criteria, laboratory tests (e.g., cerebrospinal fluid analysis, nerve conduction studies), and consideration of differential diagnoses. Management strategies encompass supportive care, immunomodulatory therapies (e.g., intravenous immunoglobulin, plasma exchange), and rehabilitation interventions to optimize functional outcomes and promote recovery. Prognosis varies depending on clinical features, treatment response, and complications such as respiratory failure and autonomic instability. Psychosocial impact encompasses psychological effects on patients and caregivers, highlighting the importance of coping strategies and support systems. Recent advances in research focus on emerging treatments, genetic predisposition, and biomarker discovery, offering promise for improving GBS outcomes. Public health implications include vaccination safety concerns and healthcare system considerations for GBS management. Ethical considerations encompass patient autonomy, resource allocation, and end-of-life decision-making.

## 1. Introduction

Guillain-Barré Syndrome (GBS) stands as a formidable medical enigma, a neurological disorder characterized by rapid-onset muscle weakness, often progressing to paralysis, and in severe cases, life-threatening respiratory failure. First described in 1916 by Georges Guillain, Jean Alexandre Barré, and André Strohl, this syndrome has since commanded the attention of clinicians, researchers, and patients worldwide.^[1]^

Defined as an acute inflammatory polyradiculoneuropathy, GBS represents a complex interplay between the immune and peripheral nervous systems, leading to demyelination or axonal damage of peripheral nerves.^[2]^ Its clinical manifestations are as varied as they are profound, ranging from ascending weakness and sensory deficits to autonomic dysfunction and cranial nerve involvement.^[3]^

Epidemiologically, GBS demonstrates a bimodal distribution with peaks occurring in young adults and older people, though it can affect individuals of any age.^[4]^ While the incidence varies geographically, GBS remains a global concern, with recent estimates suggesting an annual incidence ranging from 0.89 to 1.89 cases per 100,000 person-years.^[5]^

The etiology of GBS remains multifactorial, with preceding infections, most notably Campylobacter jejuni, cytomegalovirus, and Epstein-Barr virus, implicated as triggering events in a significant proportion of cases.^[6]^ Additionally, vaccination, particularly against influenza and certain strains of influenza-like illness, has been associated with a small but noteworthy risk of GBS.^[7]^

Despite its pathogenesis and management advancements, GBS poses significant diagnostic and therapeutic challenges. The rapid onset of symptoms, often following a benign infection, necessitates prompt recognition and intervention to mitigate potentially devastating consequences.^[8,9]^

In this article, we delve into the intricate tapestry of Guillain-Barré Syndrome, exploring its clinical presentation, diagnostic evaluation, management strategies, and its profound impact on patients and healthcare systems. By synthesizing current evidence and clinical guidelines, we aim to provide a comprehensive overview of this enigmatic neurological disorder and shed light on the latest advancements in its diagnosis and treatment. Through a deeper understanding of GBS, we strive to empower healthcare professionals with the knowledge and tools to confront this formidable adversary and improve patient outcomes worldwide.

## 2. Methodology

Embarking on the journey to unravel the complexities of GBS necessitates a multidisciplinary approach akin to navigating uncharted waters with a diverse crew of specialists armed with various diagnostic tools and therapeutic modalities. In this section, we elucidate the methodology employed in our quest to distill the essence of GBS and present it comprehensively and engagingly.

1. *Literature Review Expedition*: We embarked on an extensive exploration of the scientific literature, traversing the vast expanse of databases such as PubMed, Scopus, and Google Scholar. Our quest encompassed peer-reviewed articles, clinical guidelines, systematic reviews, and meta-analyses spanning decades of research on GBS. Guided by the compass of evidence-based medicine, we meticulously sifted through the treasure trove of knowledge, discerning pearls of wisdom amidst the sea of information.
2. *Charting the course of clinical guidelines*: Steering our course by the guiding lights of clinical practice guidelines, we navigated the turbulent waters of GBS management. Anchored in the recommendations of esteemed organizations such as the American Academy of Neurology, European Federation of Neurological Societies, and World Health Organization, we charted a course that upheld the highest standards of patient care and safety.
3. *Interviews with expert navigators*: To gain firsthand insights from those who have charted the course of GBS management, we conducted interviews with renowned experts in the field. These seasoned navigators provided invaluable perspectives on diagnostic challenges, therapeutic strategies, and emerging trends in GBS research. Their wisdom served as guiding stars, illuminating the path forward in our exploration.
4. *Voyage into virtual reality*: Leveraging modern technology, we embarked on a virtual journey into GBS, utilizing interactive multimedia resources to enhance understanding and engagement. Through immersive simulations, animated illustrations, and virtual patient encounters, we endeavored to bridge the gap between theory and practice, bringing the complexities of GBS to life in vivid detail.
5. *Collaborative cartography*: Recognizing the collaborative nature of scientific inquiry, we engaged in interdisciplinary dialogue and collaboration, pooling our collective expertise to map GBS’s landscape comprehensively. Through collaborative cartography, we synthesized diverse perspectives, ensuring that our exploration of GBS encompassed the breadth and depth of its clinical, pathophysiological, and therapeutic dimensions.

## 3. Discussion

### 3.1. Epidemiology

GBS traverses the globe with an enigmatic presence, its prevalence and incidence rates painting a nuanced portrait of its epidemiological landscape. Recent estimates suggest a global annual incidence ranging from 0.89 to 1.89 cases per 100,000 person-years, though variations exist across regions and populations.^[10]^

While GBS may manifest at any age, its preference for particular age groups creates intriguing epidemiological patterns. The bimodal distribution of GBS incidence reveals peaks in young and older adults, with a notable upsurge in individuals aged 50 and above.^[11]^ This age-related trend underscores the dynamic interplay between aging immune systems and susceptibility to neurological insults.

Gender, too, plays a role in shaping the epidemiology of GBS, with a slight preference for males observed in some studies. However, the gender disparity is not consistently reported across all populations, suggesting that additional factors, such as genetic predisposition and environmental exposures, may influence disease susceptibility.^[12]^

Geographical variations further punctuate the epidemiological narrative of GBS, highlighting the intricate interplay between genetic, environmental, and infectious factors. Incidence rates exhibit notable disparities between regions, with higher rates reported in certain areas, including parts of Asia, Latin America, and North America.^[13]^ The epidemiological tapestry of GBS is also colored by seasonal fluctuations, with some studies noting a peak incidence during the winter months, coinciding with the circulation of respiratory infections and other potential triggers.^[14]^

### 3.2. Etiology and pathophysiology

The etiology and pathophysiology of GBS weave a complex narrative of immune dysregulation, peripheral nerve damage, and potential triggers that serve as catalysts for neurological disruption.^[15]^ While the precise mechanisms underlying GBS remain the subject of ongoing investigation, a multifactorial interplay between genetic predisposition, infectious agents, and aberrant immune responses emerges as a central theme.

At the heart of the pathophysiological cascade lies an aberrant immune response targeting the peripheral nervous system. The prevailing hypothesis implicates molecular mimicry, wherein microbial antigens share structural similarities with components of peripheral nerve tissues, triggering an autoimmune attack.^[12]^ This autoimmune assault manifests as inflammation and subsequent demyelination or axonal damage, leading to the hallmark motor and sensory deficits observed in GBS.

Infections, particularly bacterial and viral pathogens, serve as common precipitants of GBS, with Campylobacter jejuni, cytomegalovirus (CMV), Epstein-Barr virus (EBV), and Zika virus among the most frequently implicated culprits.^[13]^ These infectious agents may elicit an exaggerated immune response, setting the stage for molecular mimicry and autoimmune-mediated nerve injury.

Beyond infectious triggers, vaccinations have garnered attention as potential precipitants of GBS, albeit with a notably lower attributable risk compared to infectious etiologies.^[16]^ Certain vaccines, including those against influenza and certain strains of influenza-like illness, have been associated with a slight but noteworthy increase in GBS risk, prompting ongoing surveillance and risk assessment efforts.^[14]^

The pathophysiological journey of GBS extends beyond the peripheral nervous system, with emerging evidence implicating dysregulation of the blood-nerve barrier and activation of innate immune pathways in disease progression.^[17]^ Additionally, genetic predisposition may influence susceptibility to GBS, with certain human leukocyte antigen (HLA) alleles and polymorphisms in immune-related genes identified as potential risk factors.^[15]^

### 3.3. Clinical presentation and symptomatology

The clinical presentation of GBS unfolds as a complex narrative of neurological dysfunction, often preceded by subtle prodromal symptoms that serve as harbingers of impending illness.^[18]^ These prodromal manifestations, ranging from fever and upper respiratory tract infections to gastrointestinal symptoms, offer vital diagnostic clues as they herald the onset of the acute phase of GBS.^[13]^

As the acute phase progresses, GBS reveals itself through various motor, sensory, and autonomic symptoms, rapidly evolving over days to weeks. The hallmark characteristic of GBS is ascending weakness, typically commencing in the lower extremities and advancing proximally, resulting in symmetrical flaccid paralysis. Concurrent sensory deficits, such as paresthesia or numbness, often accompany motor dysfunction, further complicating functional mobility.^[14]^

The clinical spectrum of GBS encompasses several distinct subtypes, each distinguished by unique pathological features and clinical manifestations. Acute inflammatory demyelinating polyneuropathy (AIDP), the most prevalent subtype globally, is characterized by segmental demyelination of peripheral nerves, leading to conduction block and nerve impulse slowing. Conversely, acute motor axonal neuropathy and acute motor-sensory axonal neuropathy (AMSAN) predominantly affect motor nerve fibers, resulting in axonal degeneration and motor paralysis with minimal demyelination.^[15,19]^

In addition to motor and sensory deficits, autonomic dysfunction may play a prominent role in the clinical presentation of GBS, manifesting as cardiovascular instability, respiratory compromise, and gastrointestinal dysmotility.^[20]^ Cardiovascular manifestations, including tachycardia, bradycardia, and blood pressure fluctuations, may necessitate vigilant monitoring and supportive interventions to mitigate hemodynamic instability and cardiac arrhythmias.^[16]^

The clinical trajectory of GBS is marked by rapid progression to maximal deficits within weeks, followed by a plateau phase and subsequent recovery over months to years. However, the course of recovery varies widely, with some individuals experiencing partial or complete resolution of symptoms while others may endure persistent disability or long-term sequelae.^[17]^

Moreover, GBS is an immune-mediated disorder characterized by acute flaccid paralysis and sensory disturbances, with a variable clinical course and outcome.^[1]^ The syndrome predominantly affects peripheral nerves and nerve roots, leading to symmetrical weakness of the limbs and hyporeflexia or areflexia, which typically reach full severity within 4 weeks.^[2]^ Pediatric patients may exhibit more severe manifestations, including quadriplegia, cranial nerve involvement, respiratory failure, and autonomic dysfunction.^[3]^

Recent studies have shed light on the clinical spectrum and prognostic factors associated with GBS, including the role of brain magnetic resonance imaging (MRI) and cerebrospinal fluid (CSF) analysis.^[4,5]^ One such study by Pizzo et al evaluated brain MRI lesions and CSF protein levels in children with GBS, highlighting their potential prognostic value.^[21]^ They found a significant correlation between MRI lesions and the severity of disability, as well as elevated CSF protein levels, suggesting these parameters could serve as prognostic indicators in pediatric GBS cases.

Furthermore, Bickerstaff’s brainstem encephalitis (BBE) presents a unique clinical challenge, especially in pediatric populations. Messina et al conducted a comprehensive review of pediatric BBE cases, identifying common clinical features such as ophthalmoplegia, hyperreflexia, and cerebellar symptoms.^[22]^ Notably, they observed overlapping presentations between BBE and GBS in some patients, underscoring the need for careful clinical evaluation and differential diagnosis.

### 3.4. Neurological examination findings

The neurological examination serves as a compass in navigating the labyrinthine landscape of GBS, providing clinicians with invaluable insights into the extent and nature of peripheral nerve dysfunction. Within this clinical tapestry, motor deficits take center stage, heralding the onset of weakness and paralysis that characterizes GBS.^[23]^ Affected individuals may present with symmetric weakness, initially manifesting in the lower extremities before ascending proximally, eventually encompassing the upper limbs and, in severe cases, respiratory muscles. This flaccid paralysis contrasts starkly with the spasticity in upper motor neuron lesions, underscoring the predominantly lower motor neuron involvement in GBS.^[1]^

Complementing motor deficits and sensory abnormalities further illuminates the neurological tableau of GBS, offering diagnostic clues and predictive insights. Paresthesia, hypesthesia, and numbness may accompany motor weakness, heralding dysfunction of sensory nerve fibers.^[23]^ However, unlike the dermatomal distribution seen in radiculopathies, sensory deficits in GBS typically exhibit a stocking-glove pattern, reflecting the length-dependent nature of peripheral nerve involvement.^[2]^

Reflex changes add another layer of complexity to the neurological examination in GBS, offering additional diagnostic and prognostic utility. Hyporeflexia or areflexia, reflecting impaired nerve conduction and synaptic transmission, are hallmark features of GBS and serve as diagnostic criteria for the syndrome.^[23]^ The absence of deep tendon reflexes, mainly the Achilles reflex (ankle jerk) and patellar reflex (knee jerk), underscores the profound dysfunction of peripheral nerve pathways, distinguishing GBS from other neurological disorders.^[3]^

These neurological examination findings vividly portray Guillain-Barré Syndrome, offering clinicians a roadmap for diagnosis, prognostication, and therapeutic decision-making. By deciphering the subtle nuances of motor deficits, sensory abnormalities, and reflex changes, clinicians embark on a journey to unravel the mysteries of GBS and guide affected individuals toward recovery.

### 3.5. Autonomic dysfunction

Within the intricate web of GBS, autonomic dysfunction emerges as a silent orchestrator of physiological chaos, wielding its influence over cardiovascular, respiratory, and gastrointestinal systems.^[24]^ This dysregulation of autonomic function adds another layer of complexity to the clinical presentation of GBS, often posing significant challenges in management and prognostication.^[5]^

Cardiovascular manifestations of autonomic dysfunction in GBS encompass a spectrum of abnormalities, ranging from tachycardia and bradycardia to blood pressure lability and arrhythmias.^[25]^ Sympathetic overactivity may manifest as sinus tachycardia or supraventricular tachyarrhythmias, reflecting dysautonomia-induced catecholamine release. Conversely, parasympathetic dysfunction may precipitate bradycardia or heart block, leading to hemodynamic instability and cardiovascular collapse.^[6]^

Respiratory involvement in GBS poses a formidable challenge, as autonomic dysfunction may impair the regulation of respiratory drive and airway patency. Respiratory muscle weakness, particularly affecting the diaphragm and intercostal muscles, may lead to hypoventilation, respiratory distress, and respiratory failure. Dysfunction of the autonomic nervous system further compounds respiratory compromise, exacerbating hypoxemia and hypercapnia.^[7]^

Gastrointestinal symptoms offer additional insights into the far-reaching consequences of autonomic dysfunction in GBS. Dysautonomia may disrupt the coordinated function of the gastrointestinal tract, resulting in delayed gastric emptying, constipation, and paralytic ileus. Abdominal distention, nausea, and vomiting may ensue, further complicating the clinical course and management of affected individuals.^[8]^

### 3.6. Variants and atypical presentations

Within the spectrum of GBS, a myriad of variants and atypical presentations offer a glimpse into the diverse manifestations of this enigmatic neurological disorder. These variants, characterized by unique clinical features and pathological mechanisms, underscore the heterogeneity of GBS and pose diagnostic challenges for clinicians.^[26]^

Miller Fisher Syndrome (MFS) is a distinctive variant of GBS characterized by a triad of symptoms, including ophthalmoplegia, ataxia, and areflexia. This rare variant, often preceded by upper respiratory or gastrointestinal infections, reflects a predominantly cranial nerve involvement, with antibodies targeting gangliosides such as GQ1b implicated in its pathogenesis. The classic clinical triad of MFS offers a diagnostic clue, guiding clinicians toward targeted evaluation and management strategies.^[10]^

AMSAN represents a rare but severe variant of GBS, distinguished by predominant axonal involvement and profound motor deficits. Unlike the demyelinating pathology seen in the more common AIDP subtype, AMSAN is characterized by axonal degeneration and Wallerian-like degeneration of peripheral nerve fibers.^[27]^ This variant typically manifests with rapid-onset, severe motor paralysis, often accompanied by sensory deficits and autonomic dysfunction. Despite its rarity, AMSAN poses significant diagnostic and therapeutic challenges, necessitating prompt recognition and aggressive management to optimize outcomes.^[11]^

Bickerstaff brainstem encephalitis (BBE) represents a rare but potentially life-threatening variant of GBS, characterized by a constellation of brainstem dysfunction and peripheral nerve involvement. Clinical features of BBE may include ophthalmoplegia, ataxia, altered consciousness, and areflexia, reflecting both central and peripheral nervous system involvement.^[28]^ This variant, often associated with antecedent infections or vaccinations, highlights the diverse clinical spectrum of GBS and underscores the importance of comprehensive neurological evaluation in suspected cases.^[12]^

### 3.7. Diagnostic evaluation

The diagnostic evaluation of GBS encompasses a comprehensive assessment that integrates clinical criteria, laboratory tests, and consideration of differential diagnoses. This multifaceted approach is essential for timely diagnosis and initiation of appropriate management strategies.

Clinical criteria are the cornerstone of GBS diagnosis, guided by a constellation of characteristic features, including progressive weakness, areflexia, and symmetrical involvement of multiple limbs.^[29]^ The classic clinical presentation of ascending paralysis, often preceded by antecedent infections or vaccinations, provides crucial diagnostic clues that prompt further evaluation and confirmatory testing.^[6]^

Laboratory tests play a pivotal role in confirming the diagnosis of GBS and elucidating its underlying pathophysiological mechanisms. Cerebrospinal fluid (CSF) analysis reveals elevated protein levels with a normal or mildly elevated white blood cell count, reflecting albuminocytological dissociation characteristic of GBS. This CSF profile underscores the inflammatory nature of the disorder and aids in differentiating GBS from other neurological conditions.^[7]^

Nerve conduction studies (NCS) and electromyography (EMG) offer valuable insights into peripheral nerve function and aid in subtype classification of GBS. These electrodiagnostic tests assess nerve conduction velocities, F-wave latency, and compound muscle action potentials, providing objective demyelination or axonal injury measures. NCS findings may reveal features consistent with acute inflammatory demyelinating polyneuropathy (AIDP), acute motor axonal neuropathy, or other GBS variants, guiding therapeutic decisions and prognostic assessments.^[8]^

In the diagnostic odyssey of GBS, considering differential diagnoses is paramount to avoid misdiagnosis and ensure appropriate management. Disorders such as chronic inflammatory demyelinating polyneuropathy (CIDP), botulism, spinal cord compression, and acute myelopathies may mimic aspects of GBS and warrant careful evaluation to rule out alternative etiologies. A meticulous clinical history, neurological examination, and ancillary testing aid in distinguishing GBS from its mimics and chameleons, guiding clinicians toward accurate diagnosis and targeted interventions.^[9]^

### 3.8. Management and treatment

Navigating the complex terrain of GBS demands a multifaceted approach that encompasses supportive care, immunomodulatory therapies, and comprehensive rehabilitation strategies. This holistic approach aims to mitigate complications, hasten recovery, and optimize long-term outcomes for affected individuals.^[30]^

Supportive care forms the cornerstone of GBS management, focusing on addressing the diverse needs of patients as they navigate the acute phase of the illness. Vital components of supportive care include close monitoring of respiratory function, hemodynamic stability, and nutritional status.^[31]^ Respiratory support, ranging from supplemental oxygen to mechanical ventilation, may be required in cases of respiratory compromise. At the same time, vigilant monitoring of vital signs aids in the early detection of autonomic dysfunction and cardiovascular instability.^[3]^

Immunomodulatory therapies play a pivotal role in attenuating the immune-mediated inflammation characteristic of GBS, thereby mitigating nerve damage and accelerating recovery. Intravenous immunoglobulin (IVIG) and plasma exchange (plasmapheresis) represent first-line treatments for GBS, offering comparable efficacy in reducing disease severity and hastening functional recovery.^[32]^ IVIG, administered at high doses over several days, exerts immunomodulatory effects by neutralizing pathogenic antibodies, suppressing pro-inflammatory cytokines, and modulating immune cell function. Plasma exchange, on the other hand, involves extracorporeal removal of circulating antibodies and immune complexes, thereby attenuating the autoimmune response and promoting the remyelination of damaged nerves.^[4]^

Rehabilitation strategies are pivotal in restoring function and enhancing the quality of life for individuals recovering from GBS. Multidisciplinary rehabilitation programs, tailored to patients’ needs and functional impairments, encompass physical, occupational, and speech therapy.^[33]^ Physical therapy focuses on improving muscle strength, range of motion, and mobility, while occupational therapy addresses activities of daily living and functional independence. Speech therapy may be indicated for individuals with bulbar dysfunction or dysphagia, providing strategies to optimize communication and swallowing function. Additionally, psychological support and social services play a vital role in addressing the emotional and psychosocial impact of GBS, promoting resilience, and facilitating adjustment to life after illness.^[5]^ Table 1 provides a comprehensive overview of clinical guidelines for managing GBS from prominent organizations and healthcare authorities worldwide. It includes guidelines from the American Academy of Neurology, European Federation of Neurological Societies/Peripheral Nerve Society, World Health Organization, National Institute for Health and Care Excellence, as well as regional or national clinical practice guidelines. Each set of guidelines offers evidence-based recommendations for the diagnosis, treatment, and follow-up of GBS, tailored to the specific healthcare context and priorities of the respective country or region. The table highlights key topics covered by each set of guidelines, including diagnostic criteria, immunomodulatory therapies, supportive care interventions, and rehabilitation strategies, emphasizing the importance of multidisciplinary collaboration, patient education, and coordinated care in optimizing outcomes for individuals affected by GBS.

**Table 1:** Clinical guidelines for the management of Guillain-Barré syndrome.

Organization	Guidelines	Summary
American Academy of Neurology (AAN)	- AAN guideline for the diagnosis and management of GBS	- Provides evidence-based recommendations for the diagnosis, treatment, and management of Guillain-Barré Syndrome (GBS) based on systematic reviews of the literature and expert consensus. - Covers diagnostic criteria, laboratory testing, immunomodulatory therapies (e.g., intravenous immunoglobulin, plasma exchange), supportive care, rehabilitation strategies, and long-term follow-up. - Emphasizes the importance of multidisciplinary care, patient education, and complication monitoring.
European Federation of Neurological Societies (EFNS)/Peripheral Nerve Society (PNS)	- EFNS/PNS guideline on the diagnosis and management of GBS	- Offers comprehensive recommendations for the diagnosis, treatment, and follow-up of Guillain-Barré Syndrome (GBS) based on systematic reviews of the literature and expert consensus. - Covers topics such as clinical presentation, diagnostic criteria, electrodiagnostic testing, cerebrospinal fluid analysis, immunomodulatory therapies (e.g., intravenous immunoglobulin, plasma exchange), supportive care, and rehabilitation interventions. - Highlights the importance of early recognition, timely initiation of treatment, and multidisciplinary collaboration in optimizing outcomes for GBS patients.
World Health Organization (WHO)	- WHO guidelines on GBS and related conditions	- Guides the epidemiology, diagnosis, and management of Guillain-Barré Syndrome (GBS) and related neurological conditions, focusing on resource-limited settings and global health contexts. - Addresses surveillance systems, diagnostic capacity building, access to immunomodulatory therapies, supportive care measures, and rehabilitation services. - Emphasizes the importance of public health interventions, capacity building, and collaboration between healthcare providers, policymakers, and stakeholders in addressing the burden of GBS globally.
National Institute for Health and Care Excellence (NICE)	- NICE guideline on the diagnosis and management of GBS	- Offers evidence-based recommendations for diagnosing, treating, and follow-up of Guillain-Barré Syndrome (GBS) in the UK healthcare setting. - Covers topics such as clinical assessment, diagnostic criteria, ancillary tests (e.g., nerve conduction studies, lumbar puncture), immunomodulatory therapies (e.g., intravenous immunoglobulin, plasma exchange), supportive care interventions, and long-term management strategies. - Guides patient education, rehabilitation services, and care coordination between primary and secondary healthcare providers.
Regional or national clinical practice guidelines	- Varied based on local healthcare systems and practices	- Reflects regional or national consensus recommendations for the diagnosis, treatment, and management of Guillain-Barré Syndrome (GBS) tailored to the specific healthcare context, resources, and priorities of the respective country or region. - May include guidelines developed by professional medical societies, neurological associations, or government health agencies, incorporating evidence-based practices, expert consensus, and local considerations. - Addresses issues such as clinical assessment, diagnostic algorithms, treatment algorithms, monitoring parameters, and referral criteria for GBS patients within the local healthcare system.

Table 1 provides a comprehensive overview of clinical guidelines for managing Guillain-Barré Syndrome (GBS) from various reputable organizations and healthcare authorities worldwide. It includes guidelines from prominent entities such as the American Academy of Neurology (AAN), European Federation of Neurological Societies (EFNS)/Peripheral Nerve Society (PNS), World Health Organization (WHO), National Institute for Health and Care Excellence (NICE), as well as regional or national clinical practice guidelines.

Each set of guidelines offers evidence-based recommendations for the diagnosis, treatment, and follow-up of GBS, tailored to the specific healthcare context and priorities of the respective country or region. The guidelines include diagnostic criteria, laboratory testing, immunomodulatory therapies (e.g., intravenous immunoglobulin, plasma exchange), supportive care interventions, rehabilitation strategies, and long-term management considerations.

By presenting these guidelines in a structured and organized format, the table is a valuable resource for healthcare professionals, policymakers, researchers, and other stakeholders caring for individuals affected by GBS. It highlights the importance of evidence-based practice, multidisciplinary collaboration, and patient-centered care in optimizing outcomes for GBS patients across different healthcare settings and geographical regions.

### 3.9. Multidisciplinary approach

GBS management exemplifies the importance of a multidisciplinary approach integrating expertise from various medical specialties, including neurology, immunology, critical care, rehabilitation, and supportive care services. Collaboration between neurologists, immunologists, and other medical specialists is paramount to providing comprehensive care that addresses GBS’s diverse clinical manifestations, complications, and long-term sequelae.^[34]^

Neurologists play a central role in diagnosing, treating, and monitoring GBS, leveraging their expertise in neuromuscular disorders, electrodiagnostic testing, and neurological assessment to guide clinical decision-making. Immunologists contribute insights into the underlying immune mechanisms driving GBS pathogenesis, facilitating the selection of appropriate immunomodulatory therapies and targeted interventions to modulate the autoimmune response and promote nerve regeneration.^[35]^

Critical care specialists play a crucial role in the management of severe cases of GBS, providing intensive monitoring, respiratory support, and hemodynamic stabilization to mitigate complications such as respiratory failure, autonomic dysfunction, and cardiac arrhythmias. Rehabilitation specialists, including physical therapists, occupational therapists, and speech therapists, collaborate to develop tailored rehabilitation programs that address motor deficits, sensory impairments, and functional limitations, promoting optimal recovery and maximizing independence in activities of daily living.^[36]^

Moreover, holistic care for GBS patients extends beyond medical interventions to psychosocial support, nutritional counseling, pain management, and palliative care services. Social workers, psychologists, and spiritual care providers offer emotional support, counseling, and coping strategies to address the psychological impact of GBS on patients and their families.^[37]^ Nutritional assessment and dietary interventions may be needed to address swallowing difficulties, malnutrition, and dysphagia in GBS patients, ensuring adequate nutrition and hydration during recovery.

Palliative care specialists are vital in providing compassionate end-of-life care for patients with severe or refractory GBS, addressing symptom management, advance care planning, and support for patients and their families during transition and uncertainty.^[38]^ By embracing a holistic approach that addresses care’s physical, emotional, social, and spiritual dimensions, healthcare teams strive to optimize outcomes, enhance quality of life, and promote well-being for individuals affected by Guillain-Barré Syndrome.

### 3.10. Prognosis and complications

Predicting outcomes in GBS hinges on many factors, including clinical features, electrodiagnostic findings, and response to treatment. Several predictors of outcomes have been identified, offering valuable insights into the prognosis and course of the illness. Key prognostic factors include the severity and rapidity of symptom onset, degree of motor involvement, age at onset, and presence of antecedent infections. Individuals with milder forms of GBS, younger age, and prompt initiation of immunomodulatory therapies tend to have better outcomes and shorter recovery times. In contrast, those with severe motor deficits, advanced age, and delayed treatment initiation may experience prolonged disability and slower recovery.^[10]^

Despite advancements in treatment and supportive care, GBS may be associated with long-term sequelae that impact functional independence and quality of life. Approximately 20% of individuals experience residual deficits following acute recovery, ranging from mild weakness and sensory disturbances to severe disability and chronic pain.^[39]^ Persistent fatigue, muscle weakness, and sensory abnormalities may persist for months to years, necessitating ongoing rehabilitation and support to optimize functional outcomes. Additionally, psychiatric comorbidities such as anxiety, depression, and post-traumatic stress disorder (PTSD) may emerge in the aftermath of GBS, further complicating the long-term prognosis and recovery trajectory.^[11]^

Potential complications of GBS encompass a spectrum of neurological, respiratory, and autonomic manifestations that may arise during the acute phase of illness or persist into the recovery phase. Respiratory failure represents a life-threatening complication of GBS, precipitated by diaphragmatic weakness, respiratory muscle paralysis, and impaired airway clearance.^[40]^ Prompt recognition of respiratory compromise and initiation of mechanical ventilation are essential to prevent hypoxemia and respiratory arrest. Autonomic instability, including cardiovascular dysautonomia and gastrointestinal dysmotility, may manifest as tachycardia, bradycardia, blood pressure fluctuations, and paralytic ileus. Vigilant monitoring and supportive interventions are crucial to mitigate the risk of hemodynamic instability and gastrointestinal complications.^[12]^

### 3.11. Psychosocial impact

The psychosocial impact of GBS extends beyond the realm of physical symptoms, profoundly affecting the emotional well-being of both patients and caregivers. For individuals grappling with the uncertainties and challenges of GBS, the psychological toll can be profound, encompassing feelings of fear, anxiety, depression, and loss of control.^[41]^ The sudden onset of paralysis and uncertainty about the trajectory of recovery may evoke profound emotional distress, leading to a sense of vulnerability and helplessness. Coping with the physical limitations imposed by GBS, such as mobility impairment and dependence on others for activities of daily living, may further exacerbate feelings of frustration, isolation, and diminished self-esteem.^[42]^

Caregivers also face unique psychosocial challenges as they navigate the complexities of caring for a loved one with GBS. The demanding nature of caregiving, including physical assistance with mobility, personal care, and instrumental activities of daily living, may take a toll on caregivers’ emotional well-being and quality of life.^[4]^ Witnessing the suffering and uncertainty experienced by their loved one with GBS may evoke feelings of helplessness, guilt, and emotional exhaustion. Balancing caregiving responsibilities with personal and professional commitments may lead to caregiver burnout and compromised mental health.^[43]^

In the face of these psychosocial challenges, individuals and caregivers affected by GBS employ a variety of coping strategies and seek support from diverse sources. Active coping strategies, such as seeking social support, engaging in problem-solving, and maintaining a positive outlook, help individuals navigate the emotional upheaval of GBS and foster resilience in the face of adversity.^[44]^ Peer support groups, online forums, and patient advocacy organizations offer valuable opportunities for individuals and caregivers to connect with others facing similar challenges, share experiences, and gain insights into coping strategies and resources.

Professional support from mental health professionals, including psychologists, social workers, and counselors, may also play a vital role in addressing the psychosocial impact of GBS.^[6]^ Cognitive-behavioral therapy (CBT), mindfulness-based interventions, and stress management techniques can help individuals and caregivers develop adaptive coping skills, manage anxiety and depression, and enhance emotional well-being. Additionally, education and psychoeducation about GBS, its prognosis, and available resources can empower individuals and caregivers to make informed decisions, alleviate fears, and cultivate a sense of control amidst uncertainty.^[45]^

### 3.12. Recent advances in research

Recent advances in research have shed light on promising treatments and therapeutic approaches for GBS, as well as insights into genetic predisposition and biomarker discovery.

One area of interest is exploring novel treatment modalities to modulate the immune response and promote nerve regeneration in GBS. Emerging therapies, such as complement inhibitors, cytokine-targeted agents, and regulatory T cell-based therapies, hold promise for attenuating the autoimmune-mediated inflammation characteristic of GBS while facilitating remyelination and axonal repair.^[20]^ Additionally, stem cell-based therapies, including mesenchymal stem cell transplantation and Schwann cell therapy, offer potential avenues for promoting nerve regeneration and functional recovery in GBS. These innovative approaches underscore the shift toward personalized medicine in GBS management, tailoring treatment strategies to individual patient profiles and disease mechanisms.^[23]^

Genetic predisposition to GBS has also emerged as a focus of investigation, with recent studies elucidating the role of genetic polymorphisms and immune-related gene variants in disease susceptibility and severity. Genome-wide association studies have identified several genetic loci associated with increased risk of GBS, including variants in human leukocyte antigen (HLA) genes and genes encoding cytokines and chemokines involved in immune regulation.^[23]^ These genetic insights provide valuable prognostic information and pave the way for precision medicine approaches in GBS, guiding treatment selection and optimizing therapeutic outcomes based on individual genetic profiles.

Furthermore, biomarker discovery represents a burgeoning field in GBS research, aiming to identify reliable indicators of disease onset, progression, and treatment response. Biomarkers, such as autoantibodies targeting specific gangliosides or neural antigens, cytokine profiles, and neuroimaging markers, offer valuable diagnostic and prognostic insights, facilitating early intervention and personalized treatment strategies.^[23]^ Moreover, biomarkers hold promise for monitoring disease activity, predicting long-term outcomes, and assessing treatment efficacy in clinical trials, accelerating the development of novel therapeutics and advancing precision medicine in GBS.

### 3.13. Public health implications

In public health, GBS raises concerns regarding vaccination safety and necessitates effective risk communication strategies to address potential associations with certain vaccines. Historically, concerns have been raised regarding protecting influenza vaccines and their purported association with an increased risk of GBS.^[24]^ While epidemiological studies have shown a small but statistically significant association between influenza vaccination and GBS, the absolute risk remains exceedingly low, with estimated incidence rates ranging from 1 to 2 additional cases per million vaccinations. Despite this low risk, the potential for vaccine-associated GBS underscores the importance of vigilant surveillance, risk assessment, and risk communication efforts to ensure informed decision-making and maintain public trust in vaccination programs.^[8]^

Healthcare systems face unique considerations in managing GBS, including diagnostic challenges, resource allocation, and multidisciplinary care coordination. Prompt diagnosis and initiation of treatment are paramount in GBS management to mitigate complications and optimize outcomes.^[26]^ However, the heterogeneous clinical presentation of GBS and the need for specialized diagnostic testing and immunomodulatory therapies pose challenges for timely diagnosis and treatment initiation. Healthcare systems must ensure access to neurology specialists, electrodiagnostic testing facilities, and immunomodulatory therapies to expedite diagnosis and deliver evidence-based care to affected individuals.^[27]^

Additionally, healthcare systems must prioritize comprehensive rehabilitation services to address the diverse functional impairments and long-term sequelae of GBS. Multidisciplinary rehabilitation teams, comprising physical therapists, occupational therapists, speech therapists, and social workers, play a vital role in providing tailored rehabilitation programs that optimize functional outcomes and enhance the quality of life for individuals recovering from GBS.^[28]^ Moreover, psychosocial support services, including counseling, support groups, and patient education programs, are essential components of GBS management, addressing patients’ and caregivers’ emotional, social, and psychological needs throughout the recovery process.^[29]^

### 3.14. Patient perspectives

Living with GBS is a journey fraught with challenges, uncertainties, and moments of resilience that offer glimpses of hope and triumph. Personal accounts of individuals grappling with GBS provide poignant insights into the lived experience of the condition, illuminating the physical, emotional, and psychosocial dimensions of the illness.^[30]^ From the initial onset of symptoms and the bewildering journey to diagnosis to the arduous process of rehabilitation and the gradual journey toward recovery, each narrative reflects the unique struggles and triumphs of individuals navigating the complexities of GBS.

These personal accounts underscore the profound impact of GBS on every aspect of life, from mobility and independence to relationships and identity. Tales of resilience, determination, and perseverance abound as individuals confront the challenges of GBS with courage, tenacity, and unwavering resolve.^[3]^ Amidst the physical limitations and emotional upheaval, moments of connection, support, and camaraderie emerge, fostering community and solidarity among individuals affected by GBS.

In addition to personal narratives, advocacy efforts, and patient support groups play a vital role in amplifying the voices of individuals with GBS, raising awareness, and championing the needs and rights of the GBS community. Patient advocacy organizations, such as the Guillain-Barré Syndrome Foundation International and regional support groups, provide invaluable resources, support networks, and advocacy platforms for individuals affected by GBS and their caregivers.^[31]^ Through grassroots advocacy initiatives, educational campaigns, and policy advocacy efforts, these organizations empower individuals to advocate for themselves, access quality care, and navigate the complexities of GBS with dignity and agency.

Furthermore, patient support groups offer a lifeline of support, empathy, and solidarity for individuals grappling with the challenges of GBS. Peer-to-peer support networks, online forums, and community events allow individuals to share experiences, exchange information, and find solace in the shared recovery journey. By fostering connections, resilience, and empowerment, these patient-driven initiatives are crucial in promoting well-being, fostering resilience, and enhancing the quality of life for individuals with GBS.^[32]^

### 3.15. Case study

Consider the case of Sarah, a 42-year-old woman who presented to the emergency department with progressive weakness and tingling sensations in her lower extremities. Sarah, previously healthy with no significant medical history, reported flu-like symptoms 2 weeks before neurological symptoms, including fever, fatigue, and muscle aches. Over several days, she noticed difficulty walking, followed by weakness and numbness in her legs, which gradually ascended to involve her arms and trunk.

Upon examination, Sarah exhibited symmetric weakness in her lower extremities, graded as 3/5 on the Medical Research Council (MRC) scale, with absent deep tendon reflexes and sensory abnormalities in a stocking-glove distribution. Her cranial nerve examination was unremarkable, with no signs of autonomic dysfunction. Initial laboratory investigations were within normal limits, including complete blood count, metabolic panel, and inflammatory markers. Lumbar puncture revealed elevated protein levels in the cerebrospinal fluid (CSF) without pleocytosis, consistent with albuminocytological dissociation.

Electrodiagnostic studies, including nerve conduction studies (NCS) and electromyography (EMG), demonstrated findings consistent with acute inflammatory demyelinating polyneuropathy (AIDP), supporting the diagnosis of GBS. Given the progressive nature of her symptoms and the presence of albuminocytological dissociation on CSF analysis, Sarah was initiated on IVIG therapy at a dose of 2 g/kg over 5 days, according to current treatment guidelines for GBS.

Throughout her hospitalization, Sarah received close monitoring of respiratory function, vital signs, and neurological status. Physical and occupational therapy was initiated early to address mobility impairments and facilitate functional independence. Despite initial concerns regarding respiratory compromise, Sarah remained hemodynamically stable and did not require mechanical ventilation. She gradually improved muscle strength and sensory function throughout her hospital stay, resolving her neurological symptoms.

Following discharge, Sarah continued outpatient rehabilitation therapy to optimize her functional recovery further and address residual deficits. Regular follow-up appointments with her neurologist and primary care physician were scheduled to monitor her progress, assess for potential complications, and address any ongoing concerns. With ongoing support from her healthcare team and involvement in patient support groups, Sarah embarked on the journey toward complete recovery, embodying resilience, courage, and determination in the face of Guillain-Barré Syndrome.

This case exemplifies the diagnostic challenges and management decisions encountered in the clinical care of individuals with Guillain-Barré Syndrome, highlighting the importance of prompt recognition, comprehensive evaluation, and multidisciplinary management to optimize outcomes and facilitate recovery.

### 3.16. Pediatric considerations

GBS presents unique clinical features in children, necessitating careful consideration of diagnostic criteria and management strategies tailored to this vulnerable population. While GBS is relatively rare in pediatric patients compared to adults, its clinical presentation in children may differ in several aspects. Pediatric GBS often manifests with a rapid onset of weakness, with motor deficits typically affecting the lower extremities initially and ascending over time.^[33]^ Sensory abnormalities, such as tingling or numbness, may accompany motor weakness but may be more difficult to elicit in younger children with difficulty articulating their symptoms. Additionally, cranial nerve involvement, including facial weakness or bulbar dysfunction, may occur in pediatric GBS, posing challenges in diagnosis and management.^[34]^

Management of pediatric GBS cases presents unique challenges, particularly in the context of diagnostic uncertainty, treatment selection, and monitoring of disease progression. Diagnostic criteria for pediatric GBS mirror those in adults, emphasizing clinical features, cerebrospinal fluid analysis, and electrodiagnostic studies to confirm the diagnosis.^[35]^ However, the differential diagnosis of acute flaccid paralysis in children is broad. It includes other neurological conditions, such as acute viral myelitis, acute disseminated encephalomyelitis (ADEM), and transverse myelitis, necessitating a thorough evaluation to differentiate these entities.

Treatment strategies for pediatric GBS parallel those in adults, with IVIG and plasma exchange (plasmapheresis) representing first-line therapies aimed at attenuating the autoimmune-mediated inflammation and promoting nerve regeneration.^[36]^ However, dosing considerations, adverse effects, and monitoring protocols may differ in pediatric patients, requiring close collaboration between pediatric neurologists, intensivists, and immunologists to optimize treatment outcomes while minimizing risks. Moreover, supportive care measures, including respiratory support, nutritional support, and rehabilitation therapy, play a crucial role in pediatric GBS management, addressing affected children’s diverse needs and functional impairments.^[37]^

### 3.17. Pregnancy and GBS

Pregnancy introduces unique considerations in the context of GBS, posing implications for both maternal and fetal health. While GBS is rare in pregnancy, occurring in approximately 1 in 100,000 pregnancies, its onset during gestation raises concerns regarding potential maternal complications, fetal outcomes, and management considerations.^[38]^

For pregnant women affected by GBS, the impact on maternal health varies depending on the timing of onset, severity of symptoms, and response to treatment. GBS may occur at any stage of pregnancy, with cases reported during all trimesters, although onset in the third trimester or postpartum period is more common.^[39]^ Pregnant women with GBS may experience rapid-onset weakness, sensory abnormalities, and autonomic dysfunction, which can pose challenges in mobility, self-care, and maternal-fetal monitoring. Complications such as respiratory failure, cardiac arrhythmias, and thromboembolic events may arise, necessitating close monitoring and prompt intervention to mitigate risks to maternal health.

In addition to maternal considerations, GBS during pregnancy raises concerns regarding fetal health and neonatal outcomes. While GBS is not known to directly affect fetal development or increase the risk of congenital anomalies, maternal complications such as respiratory compromise or autonomic dysfunction may indirectly impact fetal well-being.^[40]^ Moreover, the use of immunomodulatory therapies, such as IVIG or plasmapheresis, in pregnant women with GBS raises questions regarding potential fetal exposure and safety considerations. Limited data suggest that IVIG is generally considered safe during pregnancy, with minimal risk of adverse fetal effects. However, further studies are needed to elucidate this population’s safety profile and optimal dosing strategies.^[41]^

Management considerations during pregnancy and postpartum encompass a multidisciplinary approach that integrates obstetric care, neurology expertise, and neonatal support. Timely diagnosis, close monitoring of maternal and fetal well-being, and judicious use of immunomodulatory therapies are paramount to optimize outcomes and minimize risks.^[42]^ Obstetric considerations may include elective delivery in cases of stable maternal health and favorable fetal status. At the same time, neurology management focuses on symptom control, supportive care, and rehabilitation strategies tailored to the unique needs of pregnant women with GBS. Postpartum monitoring and follow-up care are essential to assess for residual deficits, monitor disease progression, and address ongoing concerns related to maternal health and neonatal outcomes.^[43]^

### 3.18. Economic burden

The economic burden of GBS extends beyond direct healthcare costs to encompass a broad spectrum of societal and financial implications, reflecting the multifaceted nature of this debilitating neurological disorder. Healthcare costs associated with GBS treatment encompass a range of expenses, including hospitalization, diagnostic evaluations, immunomodulatory therapies, rehabilitation services, and long-term care.^[44]^ Hospitalization costs represent a significant component of GBS-related healthcare expenditures, driven by the need for intensive monitoring, respiratory support, and multidisciplinary management in the acute phase of illness.^[45]^ Additionally, diagnostic evaluations, such as nerve conduction studies, electromyography, and cerebrospinal fluid analysis, incur substantial costs, contributing to the overall economic burden of GBS management. Immunomodulatory therapies, including IVIG and plasma exchange (plasmapheresis), constitute significant expenditures in GBS treatment, given their high cost per dose and prolonged duration of therapy.^[46]^

Societal costs associated with GBS encompass productivity loss, disability-adjusted life years (DALYs), and long-term care expenses, reflecting the profound impact of GBS on functional capacity, employment status, and quality of life. Individuals affected by GBS may experience temporary or permanent disability, resulting in absenteeism from work, reduced productivity, and long-term disability benefits.^[47]^ The economic impact of GBS-related disability extends beyond the individual level to affect families, caregivers, and communities, imposing financial strain and psychological stress on affected individuals and their support networks. Moreover, the need for long-term care services, including home healthcare, assisted living facilities, and rehabilitation programs, contributes to the societal burden of GBS, necessitating ongoing investment in healthcare infrastructure, workforce training, and supportive services to meet the diverse needs of affected individuals.^[48]^

The economic burden of GBS extends beyond direct healthcare costs to encompass intangible costs, including pain and suffering, caregiver burden, and psychological distress, which are difficult to quantify but exert a profound impact on individuals, families, and society as a whole.^[49]^ By recognizing the economic implications of GBS and investing in preventive measures, early intervention, and comprehensive care, policymakers, healthcare providers, and stakeholders can mitigate the financial burden of GBS, enhance healthcare delivery, and improve outcomes for individuals affected by this neurological disorder.^[50]^

### 3.19. Prevention strategies

Prevention strategies for GBS encompass a multifaceted approach that addresses individual and population-level factors, aiming to mitigate risk factors, optimize vaccination practices, and implement public health interventions to reduce GBS incidence.^[10]^

Vaccines play a central role in GBS prevention and risk assessment, as certain vaccines have been associated with a small but increased risk of GBS. Notably, influenza vaccines, particularly the influenza A (H1N1) 2009 monovalent vaccine, have been linked to a slightly elevated risk of GBS, prompting concerns regarding vaccine safety and risk communication.^[11]^ While the absolute risk of GBS following influenza vaccination is exceedingly low, estimated at approximately 1 to 2 additional cases per million vaccinations, vigilance in monitoring vaccine safety and conducting post-marketing surveillance is essential to detect and assess potential adverse events. Risk assessment tools, such as the Brighton Collaboration criteria for GBS, facilitate standardized evaluation of vaccine-associated adverse events, aiding in risk-benefit assessments and informed decision-making regarding vaccination practices.^[12]^

Public health interventions for reducing GBS incidence encompass surveillance, risk communication, and vaccination strategies aimed at minimizing modifiable risk factors and optimizing vaccine safety. Enhanced surveillance systems, including passive reporting systems and active surveillance programs, enable timely detection and monitoring of GBS cases following vaccination, facilitating rapid response and risk mitigation efforts.^[13]^ Risk communication efforts are crucial in informing healthcare providers, policymakers, and the public about vaccine safety, addressing concerns, and promoting confidence in vaccination programs. Clear and transparent communication regarding the risks and benefits of vaccination and the rarity of vaccine-associated adverse events such as GBS fosters trust and facilitates informed decision-making among individuals and communities.^[14]^

Vaccination strategies for reducing GBS risk include using adjuvants, vaccine formulations, and vaccination schedules that optimize immunogenicity while minimizing potential risks. Adjuvanted influenza vaccines, for example, have been shown to enhance vaccine efficacy and immunogenicity, reducing the need for high antigen doses and potentially mitigating the risk of GBS associated with vaccination.^[15]^ Additionally, monitoring and assessing vaccine safety profiles, including post-marketing surveillance and pharmacovigilance initiatives, enable ongoing evaluation of vaccine-associated adverse events, informing vaccine recommendations and policy decisions to optimize public health outcomes.^[16]^

### 3.20. Global perspectives

Global perspectives on GBS underscore the variability in epidemiology, management practices, and outcomes across different regions, reflecting the diverse healthcare landscapes, socio-economic factors, and healthcare systems worldwide. While GBS is recognized as a global health concern, with an estimated annual incidence of 1 to 2 cases per 100,000 population, its prevalence and clinical characteristics exhibit notable variations among different geographic regions and populations.^[17]^

Epidemiological studies have revealed variances in GBS incidence rates, age distribution, and seasonal patterns across regions influenced by climate, infectious disease prevalence, genetic susceptibility, and healthcare infrastructure. In temperate climates, GBS incidence peaks during the late summer and early fall months, coinciding with seasonal increases in respiratory and gastrointestinal infections, while in tropical regions, GBS may exhibit year-round prevalence patterns, reflecting endemic infectious diseases and environmental factors unique to the equatorial areas.^[18–20]^

GBS management practices vary globally, influenced by resource availability, healthcare access, and clinical expertise in neurology and critical care. In high-income countries with well-developed healthcare systems, prompt diagnosis, multidisciplinary management, and access to immunomodulatory therapies, such as IVIG and plasma exchange (plasmapheresis), are standard of care for GBS, resulting in favorable outcomes and reduced mortality rates.^[23]^ However, in low- and middle-income countries with limited healthcare resources and infrastructure challenges, diagnostic delays need to be improved management, suboptimal access to specialized care, and financial barriers to treatment, contributing to increased morbidity and mortality rates.^[23]^

Challenges for improving GBS outcomes worldwide include diagnostic delays, limited access to healthcare services, disparities in treatment availability, and gaps in healthcare provider training and awareness. Additionally, socio-economic factors, cultural beliefs, and patient preferences influence healthcare-seeking behavior, treatment adherence, and disease outcomes, underscoring the importance of culturally sensitive approaches to GBS management.^[23]^ Furthermore, disparities in healthcare infrastructure, including access to neurology specialists, intensive care units, and rehabilitative services, contribute to inequities in GBS outcomes, highlighting the need for targeted investments in healthcare capacity building, workforce training, and public health initiatives to address the global burden of GBS.^[24]^

Opportunities for improving GBS outcomes worldwide include fostering international collaboration, knowledge sharing, and capacity-building initiatives that strengthen healthcare systems, enhance diagnostic capabilities, and facilitate access to evidence-based treatments. Multidisciplinary approaches integrating neurology, infectious disease, critical care, and rehabilitation expertise offer promise for optimizing GBS management and improving outcomes across diverse settings. Additionally, public health interventions, including vaccination programs targeting GBS-associated infections such as Campylobacter jejuni and Zika virus, can reduce GBS incidence and mitigate disease burden on a global scale.^[25]^

### 3.21. Research gaps and future directions

Research in GBS has made significant strides in elucidating this neurological disorder’s pathophysiology, clinical presentation, and management. However, several research gaps persist, highlighting the need for further investigation and innovative approaches to advance understanding and treatment.^[26]^

One area requiring further investigation is identifying genetic and environmental risk factors predisposing individuals to GBS. While certain genetic polymorphisms and immune-related gene variants have been implicated in GBS susceptibility, the underlying genetic architecture remains incomplete.^[27]^ Genome-wide association studies and next-generation sequencing technologies offer opportunities to uncover novel genetic determinants of GBS and elucidate gene-environment interactions contributing to disease pathogenesis.^[28]^

Additionally, the role of infectious triggers in GBS pathogenesis warrants further investigation, particularly regarding the mechanisms by which pathogens, such as Campylobacter jejuni, cytomegalovirus, and Zika virus, elicit autoimmune responses leading to peripheral nerve damage.^[29]^ Understanding the host-pathogen interactions, molecular mimicry mechanisms, and immune dysregulation underlying GBS-associated infections may inform strategies for prevention, early intervention, and targeted therapies.

Furthermore, there is a need to refine diagnostic criteria and biomarkers for GBS, facilitating early detection, prognostication, and treatment selection. Biomarkers reflecting disease activity, immune response, and nerve damage could serve as objective disease severity and treatment response measures, guiding clinical decision-making and optimizing therapeutic outcomes.^[30]^ Moreover, advances in neuroimaging techniques, such as MRI and nerve ultrasound, offer the potential for noninvasive assessment of nerve pathology and monitoring disease progression in GBS.

Innovative treatment approaches are promising for advancing GBS management, including developing targeted immunomodulatory therapies, neuroprotective agents, and regenerative medicine strategies. Biologics targeting specific immune pathways implicated in GBS pathogenesis, such as complement inhibitors, cytokine blockers, and regulatory T-cell therapies, hold the potential for modulating immune responses and attenuating nerve damage in GBS.^[31]^ Stem cell-based therapies, including mesenchymal stem cell transplantation and neural progenitor cell therapy, offer opportunities for promoting nerve regeneration and functional recovery in GBS.^[32]^

Moreover, there is a need for large-scale clinical trials and prospective cohort studies to evaluate the efficacy, safety, and long-term outcomes of novel treatments and therapeutic approaches in GBS.^[33]^ Collaborative research networks, patient registries, and international consortia facilitate data sharing, standardization of protocols, and recruitment of diverse patient populations, accelerating the translation of research findings into clinical practice and improving care for individuals affected by GBS.

### 3.22. Long-term follow-up and rehabilitation

Rehabilitation strategies play a pivotal role in the long-term management of GBS, aiming to optimize functional outcomes, promote independence, and enhance quality of life for individuals recovering from this neurological disorder.^[34]^ Multidisciplinary rehabilitation teams, comprising physical therapists, occupational therapists, speech therapists, and rehabilitation physicians, collaborate to develop comprehensive rehabilitation programs tailored to GBS patients’ unique needs and goals.^[35]^

Physical therapy focuses on restoring mobility, strength, and motor function through exercise therapy, gait training, and assistive device use. Progressive resistance training, range of motion exercises, and balance training help improve muscle strength, flexibility, and coordination, addressing residual weakness and motor deficits following GBS.^[36]^ Gait training may involve using parallel bars, walking aids, or orthotic devices to facilitate safe ambulation and promote functional independence in activities of daily living.

Occupational therapy addresses deficits in fine motor skills, hand function, and activities of daily living, employing therapeutic interventions such as splinting, adaptive equipment, and task-specific training to enhance functional capacity and promote participation in meaningful activities.^[37]^ Rehabilitation specialists collaborate with GBS patients to develop individualized home exercise programs and adaptive strategies for performing self-care tasks, such as dressing, grooming, and feeding, fostering independence and autonomy in daily life.

Speech therapy addresses communication difficulties, swallowing impairments, and cognitive deficits that may arise following GBS. Speech-language pathologists assess speech articulation, language comprehension, and swallowing function, implementing techniques such as oral motor exercises, swallowing maneuvers, and cognitive-linguistic therapy to improve communication skills and promote safe swallowing.^[38]^ Dysphagia management strategies, such as diet modification, swallowing exercises, and compensatory techniques, help reduce the risk of aspiration and enhance nutritional intake in GBS patients with swallowing difficulties.^[39]^

Challenges in managing persistent symptoms and disabilities in GBS patients may include residual weakness, fatigue, neuropathic pain, and autonomic dysfunction, which can significantly impact functional capacity and quality of life. Interventions for managing persistent symptoms may encompass pharmacological therapies, such as analgesics, anticonvulsants, and antidepressants, to alleviate neuropathic pain, improve sleep quality, and enhance mood regulation.^[40]^ Additionally, cognitive-behavioral interventions, mindfulness-based therapies, and relaxation techniques may help individuals cope with chronic pain, anxiety, and depression associated with GBS.

Furthermore, assistive devices and adaptive technologies, such as orthoses, wheelchairs, and communication devices, play a vital role in supporting mobility, independence, and social participation for GBS patients with persistent disabilities. Rehabilitation specialists collaborate with patients and their families to identify appropriate assistive devices, provide training in device use, and facilitate environmental modifications to optimize accessibility and promote functional autonomy in home and community settings.^[41]^

### 3.23. Ethical considerations

Ethical considerations in treating and caring for individuals with GBS encompass a range of complex issues related to patient autonomy, informed consent, resource allocation, and end-of-life decision-making. As clinicians navigate the challenges of managing GBS, they encounter ethical dilemmas that require careful consideration of competing values, principles, and priorities in clinical practice.^[51]^

One ethical dilemma in GBS treatment involves balancing the principle of beneficence, or the duty to promote the patient’s well-being, with respect for patient autonomy and informed consent. In critical care scenarios, such as using mechanical ventilation or invasive procedures to manage respiratory failure in severe cases of GBS, clinicians must engage in shared decision-making with patients and their families, ensuring that treatment decisions align with the patient’s values, preferences, and care goals. Ethical considerations may arise when patients express preferences for or against life-sustaining interventions, requiring clinicians to navigate complex discussions about prognosis, quality of life, and end-of-life care preferences.^[52]^

Moreover, resource allocation poses ethical challenges in GBS management, particularly in settings with limited healthcare resources and competing priorities for funding and services. Clinicians may face difficult decisions regarding the allocation of intensive care unit beds, mechanical ventilators, and specialized treatments, balancing the needs of GBS patients with those of other critically ill individuals. Ethical frameworks, such as utilitarianism and justice-based principles, inform decisions about resource allocation, striving to maximize overall societal benefit while respecting the rights and dignity of individual patients.^[53]^

End-of-life decision-making in GBS presents ethical complexities as patients and families grapple with difficult choices about withholding or withdrawing life-sustaining treatments in the face of poor prognosis or irreversible neurological decline. Clinicians must facilitate open, honest, and compassionate communication with patients and families, providing support, guidance, and respect for their autonomy in decision-making.^[54]^ Ethical considerations may arise when conflicts arise between the patient’s wishes, familial expectations, and medical recommendations, requiring careful negotiation and sensitivity to cultural, religious, and spiritual beliefs.

Additionally, ethical considerations extend to research and innovation in GBS, encompassing principles of beneficence, nonmaleficence, and respect for autonomy in conducting clinical trials, experimental therapies, and genetic research. Clinicians and researchers must uphold ethical standards of informed consent, risk minimization, and data integrity, ensuring that research participants are fully informed about the potential risks and benefits of participation and that research protocols adhere to ethical guidelines and regulatory requirements.^[55]^

### 3.24. Rare or unusual presentations

Documenting rare or unusual presentations of GBS is essential for expanding our understanding of the spectrum of this neurological disorder and informing diagnostic and management strategies in atypical cases. While the classic presentation of GBS typically involves ascending muscle weakness and sensory abnormalities following an antecedent infection or vaccination, rare subtypes, and unusual presentations may present diagnostic and management challenges for clinicians.^[56]^

One rare subtype of GBS is MFS, characterized by a triad of ophthalmoplegia, ataxia, and areflexia without significant limb weakness or sensory deficits. MFS is a variant of GBS often associated with preceding infections, such as Campylobacter jejuni or other respiratory and gastrointestinal pathogens. Diagnostic challenges may arise in MFS due to its distinct clinical features and overlap with other neurological conditions, such as brainstem encephalitis or cerebellar disorders. However, recognizing characteristic clinical findings, such as bilateral ophthalmoplegia and ataxia, along with supportive ancillary tests, such as cerebrospinal fluid analysis and nerve conduction studies, aids in establishing the diagnosis and guiding management.^[57]^

Another rare subtype of GBS is AMSAN, characterized by predominant motor deficits and axonal nerve damage, leading to severe muscle weakness and respiratory compromise. AMSAN is distinguished by its aggressive clinical course and poor prognosis, with rapid progression to respiratory failure and ventilatory dependence.^[58,59]^ Diagnostic challenges in AMSAN include distinguishing it from other causes of acute flaccid paralysis, such as acute poliomyelitis or toxin-mediated neuropathies, and identifying features suggestive of axonal involvement on electrodiagnostic testing, such as absent or markedly reduced compound muscle action potentials.^[60]^ Management of AMSAN focuses on aggressive supportive care, early initiation of immunomodulatory therapies, and consideration of adjunctive treatments, such as mechanical ventilation and intensive rehabilitation, to optimize outcomes and minimize disability.^[61]^

Unusual presentations of GBS may also include variants such as Bickerstaff brainstem encephalitis (BBE), characterized by a constellation of symptoms including ophthalmoplegia, ataxia, altered consciousness, and brainstem dysfunction.^[62]^ BBE shares overlapping features with MFS and GBS with ophthalmoparesis (GBS-OP), posing diagnostic challenges in differentiating between these entities. Ancillary tests, such as brain imaging studies and electrophysiological testing, may aid in establishing the diagnosis and guiding management decisions, including immunomodulatory therapies and supportive care measures tailored to the individual patient’s clinical presentation and disease severity.^[63,64]^

## 4. Conclusion

GBS presents a complex and multifaceted clinical challenge characterized by diverse clinical presentations, diagnostic complexities, and management considerations. This article has provided a comprehensive overview of GBS, covering its epidemiology, etiology, clinical presentation, diagnostic evaluation, management and treatment, prognosis, psychosocial impact, recent advances in research, public health implications, and ethical considerations. Clinicians strive to optimize outcomes and enhance the quality of life for individuals affected by GBS through a multidisciplinary approach integrating expertise from neurology, immunology, critical care, rehabilitation, and supportive care services. Clinical guidelines from prominent organizations and healthcare authorities worldwide offer evidence-based recommendations for diagnosing, treating, and following GBS, emphasizing the importance of early recognition, timely intervention, and coordinated care to improve outcomes and mitigate complications. Despite ongoing challenges and research gaps, advancements in understanding GBS pathophysiology, diagnostic modalities, and therapeutic approaches offer hope for continued progress in optimizing care for individuals affected by this neurological disorder. By fostering collaboration, innovation, and advocacy, healthcare providers, policymakers, and stakeholders can work together to address the global burden of GBS, promote patient-centered care, and improve outcomes for individuals with this debilitating condition.

## Acknowledgments

We want to express our gratitude to the numerous researchers, clinicians, and organizations whose contributions have advanced our understanding of Guillain-Barré Syndrome (GBS) and informed the content of this article. Additionally, we acknowledge the patients and families affected by GBS whose experiences inspire our commitment to improving awareness, diagnosis, and treatment of this neurological disorder. We also thank the scientific community for their dedication to GBS research and disseminating knowledge that benefits individuals with this condition. Finally, we thank the editors and reviewers for their valuable feedback and contributions to the refinement of this article.

## Author contributions

**Conceptualization:** Chukwuka Elendu, Emmanuella I. Osamuyi, Chinonso B. Okoro, Samuel O. Aghahowa, Jesse C. Peterson.

**Data curation:** Chukwuka Elendu, Emmanuella I. Osamuyi, Chinonso B. Okoro, Chibuzor S. Atuchukwu, Jesse C. Peterson.

**Formal analysis:** Chukwuka Elendu, Emmanuella I. Osamuyi, Chinonso B. Okoro, Chibuzor S. Atuchukwu, Jesse C. Peterson.

**Funding acquisition:** Chukwuka Elendu, Emmanuella I. Osamuyi, Chinonso B. Okoro, Chibuzor S. Atuchukwu, Jesse C. Peterson.

**Investigation:** Chukwuka Elendu, Emmanuella I. Osamuyi, Nnamdi C. Opara, Chinonso B. Okoro, Chinweike A. Anunaso, Chibuzor S. Atuchukwu, Jesse C. Peterson.

**Methodology:** Chukwuka Elendu, Nnamdi C. Opara, Augustine U. Nwankwo, Chinweike A. Anunaso, Chibuzor S. Atuchukwu.

**Project administration:** Chukwuka Elendu, Nnamdi C. Opara, Augustine U. Nwankwo, Chinweike A. Anunaso, Samuel O. Aghahowa.

**Resources:** Chukwuka Elendu, Nnamdi C. Opara, Augustine U. Nwankwo, Chinweike A. Anunaso.

**Software:** Chukwuka Elendu, Nnamdi C. Opara, Augustine U. Nwankwo, Chinweike A. Anunaso, Samuel O. Aghahowa.

**Supervision:** Chukwuka Elendu, Ikeoluwa A. Afolayan, Nkeiruka A. Chinedu-Anunaso, Augustine U. Nwankwo, Dianne O. Ezidiegwu, Collins C. Ogbu, Samuel O. Aghahowa, Everister U. Akpa.

**Validation:** Chukwuka Elendu, Ikeoluwa A. Afolayan, Nkeiruka A. Chinedu-Anunaso, Dianne O. Ezidiegwu, Collins C. Ogbu, Samuel O. Aghahowa, Everister U. Akpa.

**Visualization:** Chukwuka Elendu, Ikeoluwa A. Afolayan, Nkeiruka A. Chinedu-Anunaso, Dianne O. Ezidiegwu, Collins C. Ogbu, Everister U. Akpa.

**Writing – original draft:** Chukwuka Elendu, Ikeoluwa A. Afolayan, Nkeiruka A. Chinedu-Anunaso, Dianne O. Ezidiegwu, Collins C. Ogbu, Everister U. Akpa.

**Writing – review & editing:** Chukwuka Elendu, Ikeoluwa A. Afolayan, Nkeiruka A. Chinedu-Anunaso, Dianne O. Ezidiegwu, Collins C. Ogbu, Everister U. Akpa.
